# Harmonized assessment of nutrient pollution from urban systems including losses from sewer exfiltration: a case study in Germany

**DOI:** 10.1007/s11356-021-12440-9

**Published:** 2021-01-26

**Authors:** Hong Hanh Nguyen, Markus Venohr

**Affiliations:** Department of Ecohydrology, Leibniz-Institute of Freshwater Ecology and Inland Fisheries, 12489 Berlin, Germany

**Keywords:** Wastewater management, Nutrient emission, Urban catchment model, Large scale, Leakage, Public and private sewers

## Abstract

A growing literature indicates that untreated wastewater from leaky sewers stands among major sources of pollution to water resources of urban systems. Despite that, the quantification and allocation of sewer exfiltration are often restricted to major pipe areas where inspection data are available. In large-scale urban models, the emission from sewer exfiltration is either neglected (particularly from private sewers) or represented by simplified fixed values, and as such its contribution to the overall urban emission remains questionable. This study proposes an extended model framework which incorporates sewer exfiltration pathway in the catchment model for a better justified pollution control and management of urban systems at a nationwide scale. Nutrient emission from urban areas is quantified by means of the Modelling of Nutrient Emissions in River Systems (MONERIS) model. Exfiltration is estimated for public and private sewers of different age groups in Germany using the verified methods at local to city scales, upscaling techniques, and expert knowledge. Results of this study suggest that the average exfiltration rate is likely to be less than 0.01 L/s per km, corresponding to approximately 1 mm/m/year of wastewater discharge to groundwater. Considering the source and age factors, the highest rate of exfiltration is defined in regions with significant proportions of public sewers older than 40 years. In regions where public sewers are mostly built after 1981, the leakage from private sewers can be up two times higher than such from public sewers. Overall, sewer exfiltration accounts for 9.8% and 17.2% of nitrate and phosphate loads from urban systems emitted to the environment, which increases to 11.2% and 19.5% in the case of no remediation scenario of projected defective sewer increases due to ageing effects. Our results provide a first harmonized quantification of potential leakage losses in urban wastewater systems at the nationwide scale and reveal the importance of rehabilitation planning of ageing sewer pipes in public and private sewer systems. The proposed model framework, which incorporates important factors for urban sewer managers, will allow further targeting the important data need for validating the approach at the regional and local scales in order to support better strategies for the long-term nutrient pollution control of large urban wastewater systems.

**Supplementary Information:**

The online version contains supplementary material available at 10.1007/s11356-021-12440-9.

## Introduction

Sustainable management of urban wastewater systems aims at cost-effective urban developments and avoiding of potential threats of pollutants to urban groundwater (Zoppou 2001; Thomes et al. 2019; Tscheikner-Gratl et al. 2020). Numerous investigations worldwide suggest that leakage (or exfiltration) from defective sewers in urban areas is amajor source of increased nutrients, suspended solids, and microbial pollutants in impacted groundwater (Reynolds and Barrett 2003; Wolf et al. 2012; Lee et al. 2015; Vystavna et al. 2018). It is particularly problematic when groundwater pollutants discharge to nearby surface water bodies, such as drinking water reservoirs and lakes (Eiswirth et al. 2003; Harvey and McBean 2014; Nguyen et al. 2017). Primary reasons causing sewer deteriorations over time include structural (e.g. broken joints, transversal and longitudinal cracks, fractures, and holes), operational (e.g. root intrusion, sediment accumulation, and grease build-up), and hydraulic capacity failures (Davies et al. 2001; Ennaouri and Fuamba 2013; Mohammadi et al. 2019; Balacco et al. 2020). Estimating sewer exfiltration plays an important role in problem-oriented management of urban wastewater systems (Rutsch et al. 2008; Ellis and Bertrand-Krajewski 2010; Rieckermann et al. 2010).

Sewer exfiltration models have been introduced in recent decades to support management of ageing sewer infrastructures and to predict the potential threats of water resource contamination in urban catchments (Yang et al. 1999; Karpf et al. 2009; Peche et al. 2017). The Pipeline Leakage Model (PLM) (Burn et al. 2007) was developed to estimate the water leakage for single pipe segments and showed potential to differentiate sewer exfiltration losses under different pipe and soil conditions (Ellis and Bertrand-Krajewski 2010; Wolf et al. 2006; Wolf et al. 2012). The study by Bhaskar et al. (2015) coupled groundwater-surface water model ParFlow to assess long-term impacts from exfiltration in urban systems and identified exfiltration from leaky pipes as the second main driver causing the change in subsurface storage volume. Furthermore, applying the model Gompitz allows to estimate the time-specific critical states of pipe defects and leakage in case studies in Germany (Caradot et al. 2017), Canada (Harvey and McBean 2014), Norway (Rokstad and Ugarelli 2015), and Austria (Fuchs-Hanusch et al. 2015). However, such sewer exfiltration models are often applicable only to a restricted spatial area of up to few hundred hectares or respective sewer network lengths, where the extensive input data requirements for model development can be satisfied (Karpf and Krebs 2005; Heinrich 2007; Roehrdanz et al. 2017). As pointed out by Ellis et al. (2009), outputs from small-scale analyses are hardly transferrable to larger scales because leakage data are often recorded at major defect sites with heavily deteriorated status and might cause significant over-estimation when interpolating to the whole sewer system.

Applications of existing sewer exfiltration models to large, e.g. municipality, district, or state, scales are still limited due to the problem of data shortage (e.g. Amick and Burgess 2000; Ellis and Bertrand-Krajewski 2010; Vystavna et al. 2018). As part of the Europe-wide project ‘Assessing and Improving the Sustainability of Urban water Resources and Systems’ (AISWURS), the Sewer Network Exfiltration and Infiltration model (NEIMO) (DeSilva et al. 2005) simulated the exfiltration rates from sewer leakages of different pipe sizes, ages, materials, and soil conditions at the city scale (Wolf et al. 2006; Wolf et al. 2012). The model was used to support the mapping of sewer pipe-groundwater interaction in the case study of the city of Rastatt, Germany, but the emission from this pathway was not validated separately (Wolf et al. 2012). Lee et al. (2015) and Roehrdanz et al. (2017) developed a spatial model of sewer exfiltration which incorporated important wastewater pollutants; however, the results were successfully validated only to the region of a small city of 108 km^2^ located on California’s central coast where the short-term data from groundwater observations were collected. Meanwhile, a complex 3D geological model was applied to the entire city of Bucharest, Romania, to quantitatively assess the groundwater and sewer system interaction, but focused on the main sewer pipes of diameter larger than 400 mm (Gogu et al. 2019). In other studies, roughly fixed exfiltration rates, expressed as certain percentage of pipeline wastewater flow, are commonly applied to represent the emission source from sewer exfiltration in water balance models of large urban catchments (e.g. Read 1996; Bütow et al. 2001; Nakayama et al. 2007; Ellis and Bertrand-Krajewski 2010). The question remains, though, on the significance of sewer exfiltration as an emission source in large-scale urban systems.

To help overcome some of the abovementioned issues and to provide a better solution for water emission control in large urban wastewater systems, several upscaling approaches have been proposed (e.g. Wolf and Hötzl 2007; Karpf and Krebs 2011; Peche et al. 2019). The review by Rutsch et al. (2008) states the necessity to develop a ‘standard’ defect area as an alternative parameter for site-specific exfiltration-relevant damages. A constant leakage factor was derived in the study by Karpf (2012) to provide an alternative to colmation layer parameters in the exfiltration model. Peche et al. (2017) and Peche et al. (2019) also pointed out the need for a more simplified approach to simulate hydraulic properties of colmation layer and hydraulic depth of sewer pipes when applying to up-scaled conditions and proposed the terminology ‘hydraulic disconnection’ of leaky pipes from groundwater for modelling sewer exfiltration in large-scale studies. Other solutions to model the ‘susceptible’ sewer exfiltration areas in large urban systems include the following: applying GIS tools to simulate the spatial-temporal patterns of sewer defect distributions (e.g. Vystavna et al. 2018), developing leakage indicators to provide evidence for the presence of sewage in shallow groundwater (e.g. Dvory et al. 2018), and conducting surveys to construct the pattern of sewer exfiltration in private sewer systems where monitoring data are even more deficient in comparison to public sewers (e.g. Ducci 2018). It is thus important to test the applicability of these solutions in order to develop the modelling framework to support management decisions of large urban wastewater systems.

This study adapts advanced approaches in sewer exfiltration modelling over the recent decades, with the primary aim to test a model framework at the nationwide scale which integrates sewer exfiltration in the catchment model in order to evaluate the potential contribution of this source in the overall emission from urban areas. With regard to the emission from wastewater leakage, the study focuses on the following specific objectives: (1) to estimate the potential magnitude and the spatial distribution of sewer exfiltration at the national scale; (2) to quantify the contribution of different sources of public and private ageing pipes to the nationwide emission of sewer exfiltration; (3) to estimate the nutrient loads from the sewer exfiltration in large urban systems and to predict the potential effects of non-compliance of remediation to ageing defective sewers by means of scenario analyses. Establishment of a general modelling framework at the nationwide level, which incorporates important factors for urban sewer managers, will assist in better targeting the data need and verification at regional and local scales to support better strategies for the long-term management of urban wastewater systems.

## Materials and methods

### Study area

**Fig. 1:**
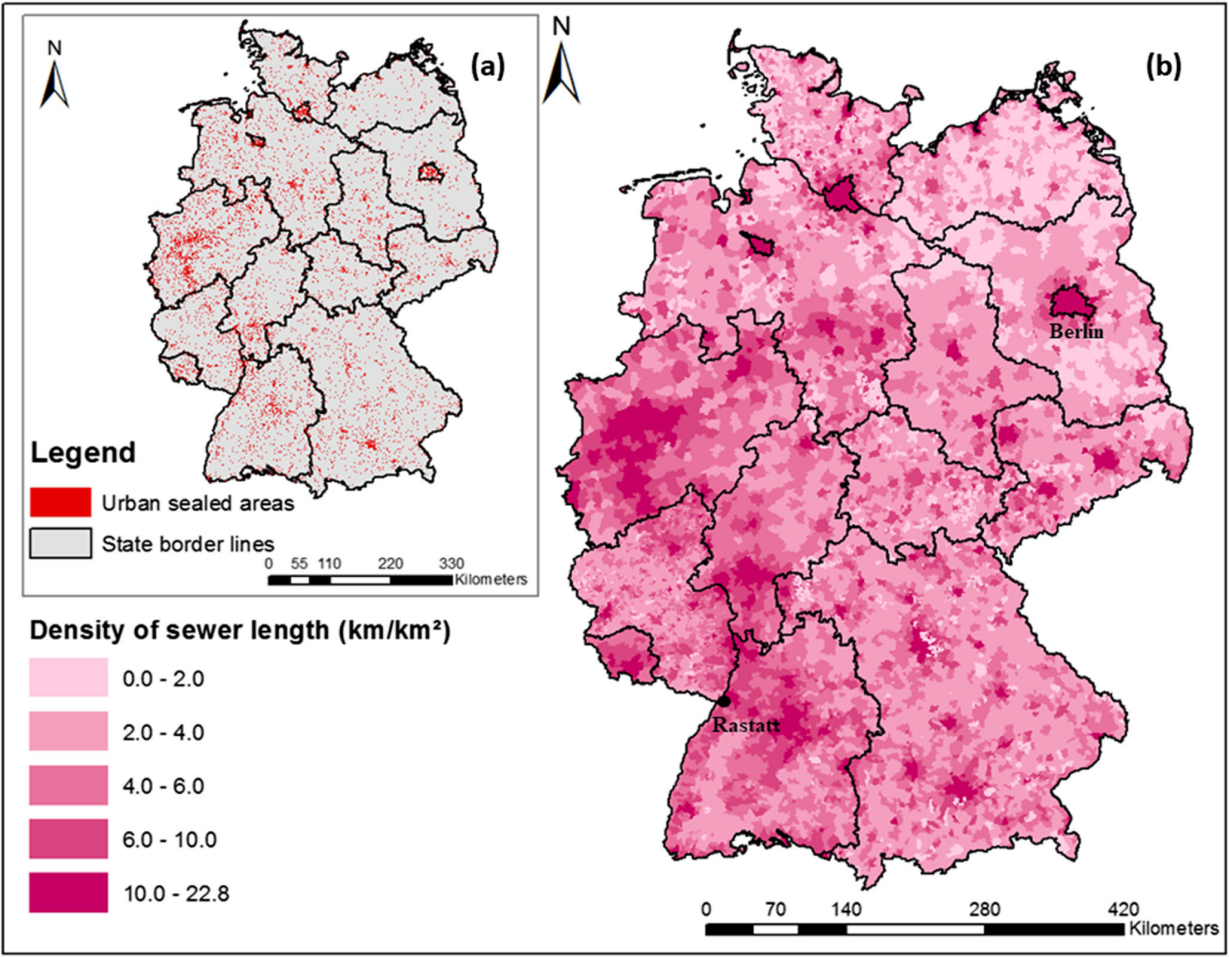
Maps of the study area: (a) sealed urban areas and (b) density of sewer pipe length per municipality units in Germany

The study applies to urban areas in Germany (Fig. 1). Germany was selected as a case study, since dominant data and methods on sewer exfiltration at various spatial and temporal scales are reported for this country (e.g. Selvakumar et al. 2004; Chisala and Lerner 2008; Ellis and Bertrand-Krajewski 2010). Table 1 reviews studies which are operated on real sewer networks of cities in Germany rather than under the condition of small-scale laboratory or field experiments. Results from studies indicate that significant increases of toxic pollutants in groundwater wells in Germany might be associated with anthropogenic sources from urban sewage or dumps (e.g. Burn et al. 2007; Wolf et al. 2012; Meinikmann et al. 2015). According to the German Association for Water, Wastewater and Waste (DWA), approximately 20% of sewers nationwide are found to be sufficiently defective to allow exfiltration to occur and in need of short to mid-term rehabilitation (Decker and Risse 1993; Berger et al. 2015). The average cost to complete the necessary repairs for the whole public sewer systems in Germany falls around 100 billion dollars (Selvakumar et al. 2004). Meanwhile, knowledge on the condition of private sewer damages is low, with less than 10% of sewers being sighted annually (Berger et al. 2015). Overall, to our knowledge, no study exists on country-wide scale evaluating the spatial distribution of sewer exfiltration from defective sewer systems; thus, emissions to the surrounding environment and groundwater from this source in urban systems remain largely unknown (Berger et al. 2015).

**Table 1:** Studies on sewer exfiltration from real sewer networks in some cities in Germany

City	Total area	Sewer length	Exfiltration rate		Study
	(km^2^)	(km)	(L/s/km)	(m^3^/cm^2^/day)	
Hannover	84	1320–2300	1.4 × 10^−4^-0.3	-	Härig and Mull (1992), Eiswirth and Hötzl (1997)
Dresden	n/a	600–1620	1.16 × 10^−5^–1.4 × 10^−3^	-	Karpf and Krebs (2005), Karpf and Krebs (2011)
Rastatt	59	208–442	2.1 × 10^−4^	-	Eiswirth (2002), Eiswirth et al. (2003), Wolf et al. (2004), Wolf et al. (2006), Wolf et al. (2012)
Dresden-Salzb	-	9.3	-	0.48	Ellis and Bertrand-Krajewski (2010)
Berlin	-	7.7–12.5	-	0.007 – 0.54	Ellis and Bertrand-Krajewski (2010)

### Input data

In this study, the main input data for the development of the sewer exfiltration model include sealed urban areas, total connected population, sewer network conditions, wastewater volume and loads, and number of effective days of stormwater events per year (Table 2). The smallest area covering data source for Germany is the municipality scale, and this was selected for our modelling. Municipality areas and boundaries are provided by the Federal Agency for Cartography and Geodesy using the 1:250,000 resolution map (VGE250), while the sealed areas were extracted from the gridded land use and land cover map for Germany (LBM_DE2015, 10 × 10 m) provided by the Federal Agency for Cartography and Geodesy (LBM 2018). Current data from the Federal Statistical Office (FDZ) is available on the connected population to sewers and public sewer attributes (total length, construction year, and sewer types) of 11,379 municipalities in Germany. The public sewer system includes rainwater sewers, separated black water sewers (BW), and combined sewers (CS, collecting rain and black water in a single pipe system), of which BW and CS sewers contribute the total pipe length of approximately 450,000 km. Regarding the age of public sewers, data from the FDZ source is available for time periods before the year 1960, 1961–1970, 1971–1980, 1981–1990, 1991–2000, 2001–2010, and after 2010. Wastewater amounts and loads from households and connected sealed urban areas as well as the average effective number of stormwater event days per year are derived from the MONERIS model based on inputs of connected inhabitants, rainfall, sealed urban areas, average volume and concentration of wastewater generated per inhabitant, and size of combined sewer storage volume (Schmidt et al. 2020).

**Table 2:** Input data for estimating sewer exfiltration of urban wastewater systems in Germany

No.	States	Sealed area (km^2^)	Connected inhabitant* (Inh.)	Length_Public sewer (km)*				Wastewater amount (m^3^/year)			Stormwater events (days/year)
				CS_bf1980	CS_af1981	BW_bf1980	BW_af1981	Private	Public_CS	Public_BW	
1	Brandenburg	792	2,366,891	373	243	1647	13,804	93	21	82	9
2	Berlin	214	3,393,130	1712	253	3645	1897	141	73	88	7
3	Baden-Württemberd	1880	10,556,277	24,996	25,165	2944	9622	449	1059	89	25
4	Bavaria	2825	12,232,141	27,469	27,770	5895	24,568	578	1343	143	27
5	Bremen	82	654,494	543	259	783	320	29	23	17	12
6	Hesse	1071	6,012,550	12,812	16,435	1703	2743	273	570	45	14
7	Hamburg	137	1,738,562	812	440	1629	904	88	57	50	13
8	Mecklenburg-Vorpommern	456	1,436,107	326	274	1141	9862	55	17	46	10
9	Lower Saxony	1853	7,365,844	1607	1751	13,634	33,185	340	84	299	18
10	North Rhine-Westphalia	2849	17,229,307	25,698	20,663	11,015	17,852	839	1368	266	24
11	Rhineland-Palatinate	774	3,977,499	12,061	9939	1314	4890	172	361	31	18
12	Schleswig-Holstein	614	2,667,195	457	1160	5633	7408	126	39	113	22
13	Saarland	187	988,532	4771	1846	286	300	41	106	6	20
14	Saxony	899	3,751,945	4811	5149	855	10,619	119	244	42	16
15	Saxony-Anhalt	648	2,155,467	1228	2212	649	11,850	73	91	40	8
16	Thuringia	538	2,025,486	3005	6604	356	3282	65	181	17	14

*CS,* combined sewer; *BW*, black water from separate sewers; *bf*, before; *af*, *after*

* Source: Research Data Centre of the Federal Statistical Office and Statistical Offices of the Federal States, [Erhebung über die öffentliche Abwasserentsorgung und Abwasserbehandlung], [survey year 2013], own calculations

The statistical information on conditions of private sewers, i.e. the connection from houses to a public sewer system, at the national scale is not available from spatially differentiated surveys in Germany (Ellis and Bertrand-Krajewski 2010; Berger et al. 2015). However, it is approximated that the total length of private sewers is around 1.1 million kilometres (Berger et al. 2015). In this study, we assume a similar ratio of connected inhabitant per sewer pipe length per municipality for both private and public sewers, respectively, since the development of sewer infrastructure in urban systems, either private or public sewers, is often accompanied with the increase of number of inhabitants connected to these sewers. Data from FDZ is extracted to derive the function of public sewer length per connected inhabitant versus connected inhabitant density per municipality, which is then applied to private sewers. The function is further adjusted to reach the total length of private sewers as reported by Berger et al. (2015) in order to estimate the details of private sewer length for each municipality (Supplementary document S1, Fig. A).

### Catchment model

The semi-empirical, conceptual model MONERIS (Venohr et al. 2011) is applied in this study to estimate the nutrient emissions to major water bodies in Germany as part of the project ‘Agro-Environmental measures in Germany’ (AGRUM-DE) (Schmidt et al. 2020). The model allows quantifying emission from seven main pathways (including atmospheric deposition, urban areas, point sources, overland flow, erosion, tile drainage, and groundwater) and has been successfully applied at various meso- to macro-scales in Germany, Europe, and other countries worldwide (e.g. Xu 2004; Von Sperling and Behrendt 2007; Venohr et al. 2010; Schmidt et al. 2020). The water component and the nutrient concentration are calculated and validated for each pathway in the model separately, and the results are lumped to the in-stream retention for final load calculation and calibration with observed gauging station data (Venohr et al. 2011).

**Fig. 2:**
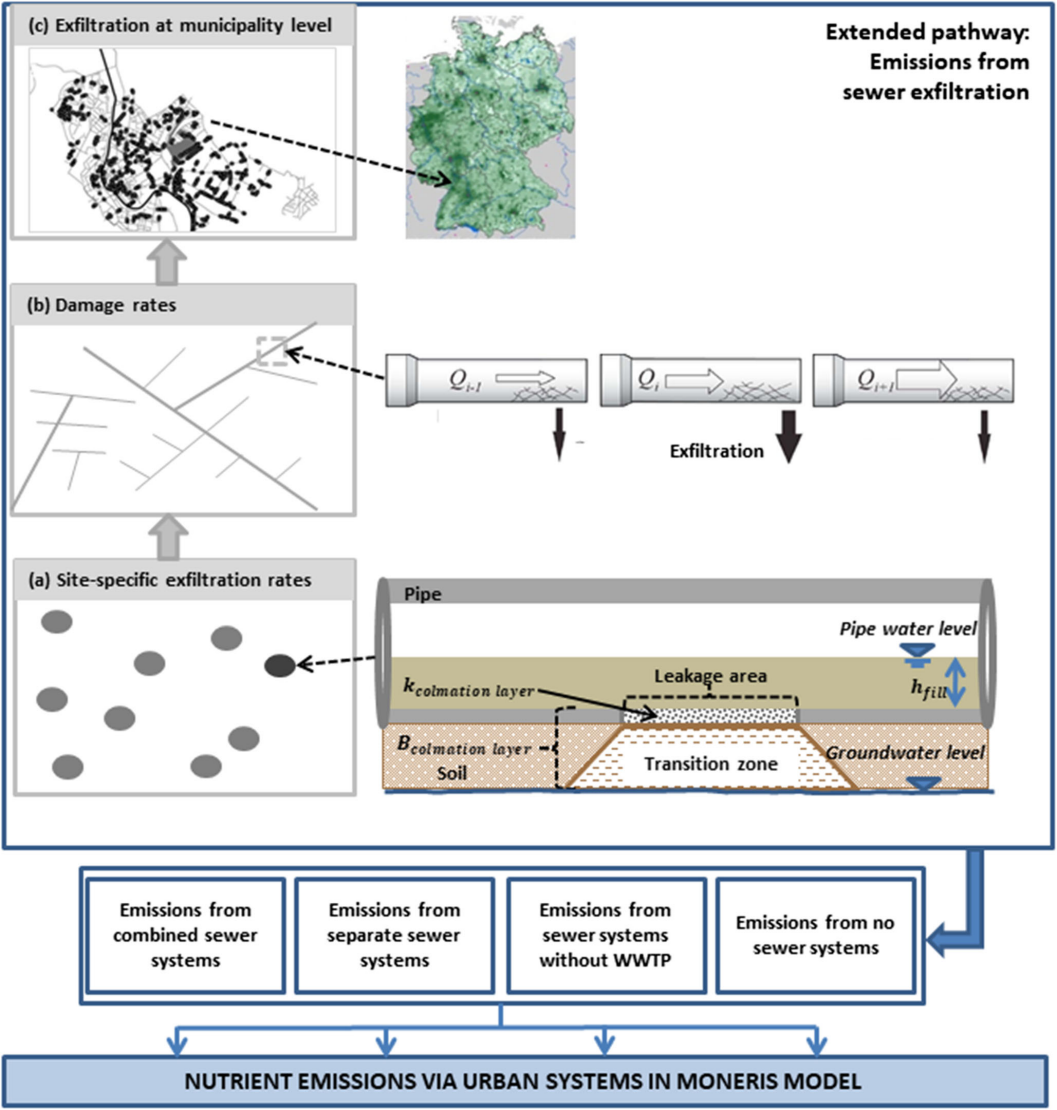
Conceptual diagram of extended pathway on sewer exfiltration in the MONERIS model for nutrient emissions in urban systems

The conceptual diagram for the extended model framework integrating the sewer exfiltration calculations in the catchment model at the national scale is provided in Fig. 2. In the extended model framework, the MONERIS model quantifies the nutrient loads from sealed urban areas and households (Schmidt et al. 2020). The mean long-term annual loads of wastewater from MONERIS provide inputs for the calculation of sewer exfiltration from urban sewers, and the validated outputs from the leakage sewer sources are integrated in the output nutrient load calculations of the urban emission pathway of the MONERIS model (Fig. 2). More details on the calculation of inputs from MONERIS for the sewer exfiltration model are provided in Supplementary document S2.

### Sewer exfiltration model

#### Estimation of exfiltration rates at single defects

The calculation of exfiltration amount at single sites to the pipe scale is based on Darcy’s law and incorporates the effects of the colmation layer (DeSilva et al. 2005; Rutsch et al. 2008; Boukhemacha et al. 2015):

1 $$ {\mathrm{Q}}_{\mathrm{ex}}={\mathrm{A}}_{\mathrm{leak}}\ast \mathrm{h}\ast \frac{{\mathrm{k}}_{\mathrm{f}}}{\mathrm{B}}\ast \mathrm{86,400,000.0} $$

where:

Q_ex_: exfiltration rate (L/day),
A_leak_: leakage area (m^2^),
h: wastewater level in the pipe (m),
k_f_: hydraulic conductivity of the clogging layer (m s^−1^), and
B: thickness of the colmation layer (m).

In Eq. (1), the ‘standard’ leakage area parameter is used alternatively to the ‘leakage area’ A_leak_ for both private and public sewers. This is based on the assumption that similar pipe characteristics and similar defective conditions induce similar exfiltration rates (Franz and Krebs 2005; Karpf 2012). The exfiltration rates are specified for conditions of dry weather flow (dwf) and stormwater flow (swf) in case of CSs, while only dwf is considered for BW and private sewers. In Eq. (1), the initial values of four parameters A_leak_, h, k_f_, and B are adjusted in the range of published data of studies mostly from Germany (particularly from the City of Rastatt, in which more than 90% of sewer networks were intensively inspected over the period of 10 years) to derive the exfiltration volumes Q_ex_ of public and private sewers (expressed in L/day unit) (Wolf et al. 2006; Wolf et al. 2012).

Due to the strong variability and thus uncertainty of the hydraulic conductivity parameter *k*_*f*_, the alternative parameter ‘leakage factor’ *K*_*L*_ is proposed for the colmation layer property (Eq. 2). This factor incorporates the two parameters hydraulic conductivity and thickness of the colmation layer, which are difficult to determine and often unknown in practice (Karpf et al. 2009; Peche 2019). This leads to the reformulation of Eq. 1 for calculation of exfiltration rates under the swf condition (which is the case of CSs in this study):

2 $$ {\mathrm{K}}_{\mathrm{L}}=\frac{{\mathrm{k}}_{\mathrm{f}}}{\mathrm{B}} $$

where:

K_L_: leakage factor (s^−1^),
k_f_: hydraulic conductivity of the clogging layer (m s^−1^), and
 B: thickness of the colmation layer (m).

3 $$ {\mathrm{Q}}_{\mathrm{ex}}={\mathrm{A}}_{\mathrm{leak}}\ast \mathrm{h}\ast {\mathrm{K}}_{\mathrm{L}} $$

#### Estimation of sewer damage rates

Next, the sewer damage rate is defined by assuming that the total area of exfiltration is proportional to the pipe damage length, i.e. the rates (or density) of damage expressed as the number of defects with sewer leakage per unit of sewer pipe length, as suggested by various studies in Germany (e.g. Rutsch 2006; Franz and Krebs 2005; Chisala and Lerner 2008).

The data on the number of defects per unit of sewer pipe length is estimated from available literature and expert knowledge. For public sewers in Germany, the average damage rate of approximately 60 defects per km was reported in the study by Decker (1994). In the AISWURS project, damage rates of less than 10 to approximately 90 defects per km are estimated for various age and sewer joint groups from cities in Europe (e.g. DeSilva et al. 2005; Chisala and Lerner 2008; Ellis and Bertrand-Krajewski 2010). Meanwhile, intensive investigations in the city Rastatt, Germany, identified 31,006 defects for 90% of inspected pipes, of which 5295 major leaks were recorded for more detailed inspection of 164 km sewer length (Wolf et al. 2006; Held et al. 2005; Wolf et al. 2012). This corresponds to an average rate of 32 to 78 leaking defects per km of pipe. Compared to public sewers, private sewers are often subject to more external impacts (e.g. from tree roots or less protection from street coverage) and less inspection, resulting in elevated damage densities and exfiltration risks (Held et al. 2005; Berger et al. 2015). According to the studies by DWA and Krönlein et al. (2015), the damage rate of private sewers can be from 25 to 75% based on available records of inspected private pipes. A study by Schleyer et al. (1991) estimated that 64 to 74% of 620,000 km of private sewers are sufficiently defective to allow exfiltration to occur. Based on these literatures, the value of 60 defects per km of sewer length is assumed as an initial default of the damage rate for public sewers (D_pub_), which include both CS and BW sewers. Meanwhile, the number of defective private sewers is assumed to be roughly three times higher than those of public sewers in order to represent the extreme case of damage densities from literature (e.g. Schleyer et al. 1991; Berger et al. 2015).

Furthermore, the effect of ageing pipes is taken into account for the D_pub_ parameter, since it is considered a primary source of sewer exfiltration in Germany (e.g. Davies et al. 2001; Berger et al. 2015; Krönlein et al. 2015). Sewer ages are divided into two major groups in terms of the date of construction based on the study by Krönlein et al. (2015), which defined the statistically significant correlation between damage rate and age factors for pipes of more than 40 years at the municipality scale. Thus, the damage factor α_i_ (i = before 1980, after 1981) for Germany is derived in this study from the regression equation of the study by Krönlein et al. (2015), with an adjustment to the mean damage rate of 20% for all sewers according to the status information for public sewers from DWA (Berger et al. 2015) (Supplementary document S1, Fig. B). In particular, the derived damage factors used for the two age groups in this study are defined as α_before 1980_ =3/2 and α_after 1981_ = 1/4, respectively (Eq. 4):

4 $$ {\mathrm{D}}_{\mathrm{pub}\_{\upalpha}_{\mathrm{i}}}={\upalpha}_{\mathrm{i}}\ast {\mathrm{D}}_{\mathrm{pub}} $$

where:

$$ {\mathrm{D}}_{\mathrm{pub}\_{\upalpha}_{\mathrm{i}}} $$
number of defects per km length of the public sewers, specified for the age group i (km^−1^),
D_pub_: number of defects per km length of the public sewers (km^−1^), and
α_i_: damage factor for the sewer age group i (unitless).

#### Estimating exfiltration volume at the municipality scale

Finally, the exfiltration rate at the municipality scale (*n* = 11,379) is quantified based on the following equations:

5 $$ {\mathrm{Q}}_{{\mathrm{ex}}_{\mathrm{n}}}={\mathrm{Q}}_{\mathrm{ex}\_{\mathrm{priv}}_{\mathrm{n}}}+{\mathrm{Q}}_{\mathrm{ex}\_\mathrm{pub}\_{\mathrm{CS}}_{\mathrm{n}}}+{\mathrm{Q}}_{\mathrm{ex}\_\mathrm{pub}\_{\mathrm{BW}}_{\mathrm{n}}} $$

where:

6 $$ {\mathrm{Q}}_{\mathrm{ex}\_{\mathrm{priv}}_{\mathrm{n}}}={\mathrm{L}}_{{\mathrm{priv}}_{\mathrm{n}}}\bullet {\mathrm{D}}_{{\mathrm{priv}}_{\mathrm{n}}}\bullet {\mathrm{Q}}_{\mathrm{ex}\_\mathrm{priv}\_{\mathrm{dwf}}_{\mathrm{n}}}\bullet 365/1000 $$

7 $$ {\mathrm{Q}}_{\mathrm{ex}\_\mathrm{pub}\_{\mathrm{CS}}_{\mathrm{n}}}={\mathrm{L}}_{\mathrm{pub}\_\mathrm{cs}\_{\upalpha_{\mathrm{i}}}_{\mathrm{n}}}\bullet {\mathrm{D}}_{\mathrm{pub}\_\mathrm{cs}\_{\upalpha_{\mathrm{i}}}_{\mathrm{n}}}\bullet {\mathrm{Q}}_{\mathrm{ex}\_\mathrm{pub}\_{\mathrm{dwf}}_{\mathrm{n}}}\bullet \left(365-\upbeta \bullet {\mathrm{SWE}}_{\mathrm{pub}\_{\mathrm{cs}}_{\mathrm{n}}}\right)/1000+{\mathrm{L}}_{\mathrm{pub}\_\mathrm{cs}\_{\upalpha_{\mathrm{i}}}_{\mathrm{n}}}\bullet {\mathrm{D}}_{\mathrm{pub}\_\mathrm{cs}\_{\upalpha_{\mathrm{i}}}_{\mathrm{n}}}\bullet {\mathrm{Q}}_{\mathrm{ex}\_\mathrm{pub}\_{\mathrm{swf}}_{\mathrm{n}}}\bullet \left(\upbeta \ast {\mathrm{SWE}}_{\mathrm{pub}\_{\mathrm{cs}}_{\mathrm{n}}}\right)/1000 $$

8 $$ {\mathrm{Q}}_{\mathrm{ex}\_\mathrm{pub}\_{\mathrm{BW}}_{\mathrm{n}}}={\mathrm{L}}_{\mathrm{pub}\_{\mathrm{BW}}_{\mathrm{n}}}\bullet {\mathrm{D}}_{\mathrm{pub}\_{\mathrm{BW}}_{\mathrm{n}}}\bullet {\mathrm{Q}}_{\mathrm{ex}\_\mathrm{pub}\_{\mathrm{dwf}}_{\mathrm{n}}}\bullet 365/1000 $$

9 $$ {\mathrm{W}}_{{\mathrm{loss}}_{\mathrm{n}}}={\mathrm{Q}}_{{\mathrm{ex}}_{\mathrm{n}}}/\left({\mathrm{W}\mathrm{W}}_{{\mathrm{priv}}_{\mathrm{n}}}+{\mathrm{W}\mathrm{W}}_{\mathrm{pub}\_{\mathrm{CS}}_{\mathrm{n}}}+{\mathrm{W}\mathrm{W}}_{\mathrm{pub}\_{\mathrm{BW}}_{\mathrm{n}}}\right) $$

In the abovementioned equations, the overall exfiltration ${\mathrm{Q}}_{{\mathrm{ex}}_{\mathrm{n}}}$ (m^3^/year) is the sum of exfiltration rates from private sewers ${\mathrm{Q}}_{\mathrm{ex}\_{\mathrm{priv}}_{\mathrm{n}}}$(m^3^/year) and public sewers (m^3^/year) (including combined sewers ${\mathrm{Q}}_{\mathrm{ex}\_\mathrm{pub}\_{\mathrm{CS}}_{\mathrm{n}}}$and separate sewers${\mathrm{Q}}_{\mathrm{ex}\_\mathrm{pub}\_{\mathrm{BW}}_{\mathrm{n}}}$). Equations (6) and (8) describe exfiltration rates of private sewer ${\mathrm{Q}}_{\mathrm{ex}\_{\mathrm{priv}}_{\mathrm{n}}}$ (m^3^/year) and public separate sewer${\mathrm{Q}}_{\mathrm{ex}\_\mathrm{pub}\_{\mathrm{BW}}_{\mathrm{n}}}$ (m^3^/year) defined as function of sewer length L (km), the damage rates D (defect/km), and the rate of exfiltration of single defects Q_ex _ dwf_ (L/day). For CSs, the exfiltration in Eq. (7) is the sum of exfiltration during dwf and swf, which takes into account the effective number of stormwater days SWE (days/year) and the storm event duration factor β. ‘Effective’ number of stormwater days refers to the total number of days on which, under consideration of precipitation, connected sealed area and storage volume, overflow events occur in CSs (Venohr et al. 2011). The rate of water loss W_loss _ (%) due to exfiltration from each sewer source is the ratio between the amount of exfiltration ${\mathrm{Q}}_{{\mathrm{ex}}_{\mathrm{n}}}$ (m^3^/year) from that source and the amount of wastewater generated from all three sources per municipality (m^3^/year). Finally, the nutrient loads of sewer exfiltration are obtained by multiplying the W_loss _ (%) with emission coefficients for each type of private sewers, CSs, and BWs, respectively.

#### Model validation and evaluation

In this study, the proposed approach for sewer exfiltration estimation is shown through stepwise equations (Eqs. 1 to 9). The interpretation of the model results based on this approach depends on model input data and parameters (Table 2 and Table 3). On large scales (e.g. federal states, municipality, or country scales), no information on the spatial distribution of sewer exfiltration is available. The study is thus relying on literature information, upscaled approach data, and expert consultations to derive the preliminary values of model parameters and variables for the proposed model framework, which can be further tested at various intermediate scales. Firstly, the literature data of parameters published from sewer studies is applied to the sewer exfiltration equations (Table 3). This allows validating if the simulated input parameters and the resulting output variables are within the practical and valid ranges of observed sewer exfiltration data. In this step, known mean values of important key parameters of sewer exfiltration are used in preference for our calculations (when applicable). This step was conducted for estimating the two main variables, i.e. exfiltration volumes and damage densities of a certain pipe length. Next, the exfiltration volumes at the municipality level are quantified and further adjustments to model parameters are reconsidered (if needed) to obtain the good agreements of the final municipality’ scale exfiltration volumes with the reported data for Germany (e.g. Ellis et al. 2009; Ellis and Bertrand-Krajewski 2010). In this regard, the final results incorporate the knowledge of important driving factors affecting sewer leakage, such as colmation layer and hydraulic conductivity (also known as coefficient of permeability), and match with the limited published data ranges for large urban scales, which helps to reduce the uncertainty for management decisions of urban wastewater systems.

**Table 3:** Parameters for sewer exfiltration quantification at the municipality scale

STT	Parameters	Abbreviation	Units	Reference data	Data sources
(a)	Exfiltration rate per defect				
1	Private sewer_dry weather flow	Q_ex_priv_dwf_	L/day	0.6–10.0	Dohmann et al. (1999), Forschergruppe (2002), Held et al. (2005), Wolf and Hötzl (2007)
2	Public sewer_dry weather flow	Q_ex_pub_dwf_	L/day		
3	Public combined sewer_stormwater flow	Q_ex_pub_swf_	L/day	< 230	Held et al. (2005), Wolf and Hötzl (2007)
	- Standard leakage area	A_leak_	m^2^	0.0015–0.018	Rauch and Stegner (1994), Wolf et al. (2006), Wolf et al. (2012)
	- Wastewater level in the pipe	h	m	0.01–0.6	Wolf and Hötzl (2007), Peche et al. (2017)
	- Colmation layer thickness	B	m	0.01–0.05	Vollertsen and Hvitved-Jacobsen (2003), Ellis et al. (2009), Karpf (2012)
	- Hydraulic conductivity	k_f_	m/s	3.0 × 10^−8^–1.0 × 10^−4^	Rauch and Stegner (1994), Dohmann et al. (1999), Vollertsen and Hvitved-Jacobsen (2003), Wolf and Hötzl (2007)
	- Leakage factor	K_L_	1/s	0.001–0.01	Rauch and Stegner (1994), Karpf and Krebs (2005), Karpf and Krebs (2011)
(b)	Damage area				
4	Private sewer	D_priv_	km^−1^	n/a	Schleyer et al. (1991), Berger et al. (2015)
5	Public sewer_Stormwater	D_pub_CS_	km^−1^	54–78	Decker (1994), DeSilva et al. (2005), Held et al. (2005), Wolf et al. (2006)
	- Sewers constructed before 1980	D_pub_CS_bf1980_			
	- Sewers constructed after 1981	D_pub_CS_af1981_			
6	Public sewer_Blackwater	D_pub_BW_	km^−1^		
	- Sewers constructed before 1980	D_pub_BW_bf1980_			
	- Sewers constructed after 1981	D_pub_BW_af1981_			
(c)	Exfiltration volume at the municipality scale				
7	Exfiltration rate	Q_ex_	L/s/km (%)	< 0.01–0.2	Hoffman and Lerner (1992), Ellis et al. (2009)
	- Private sewers	Q_ex_priv_	L/s/km (%)	n/a	
	- Public sewers	Q_ex_pub_	L/s/km (%)	0.011–2.0	Schleyer et al. (1991), Hoffman and Lerner (1992), Ellis and Bertrand-Krajewski (2010)

Besides, in order to evaluate the importance of input data for the model building process, the simple linear regression analysis is performed on main input data (Table 2). Linear regression models are a simple but effective method to define the relationships of model inputs and outputs, which evaluate how the variation of a single input can affect the interpretation of the model output, while other inputs are kept unchanged (Hamby 1994; Saltelli et al. 2006). This sensitivity analysis helps to define the most important model parameters based on the nationwide database of sewers in Germany and provides the background for more spatially explicit studies in the future at finer scales. The linear regression is applied in the model using lm () function in R version 3.6.3.

### Scenario analysis

Based on the established model framework, the contribution of sewer exfiltration source in the overall nutrient emission of urban areas at the nationwide scale is quantified. Besides, the model is tested against a set of altered inputs in order to identify the effects of shortage of sewer emission control on urban system management. The validated model results serve as baseline scenario (BS); meanwhile, the scenario of non-appliance of remediation to the predicted 20% defective sewers, which are in need of short to mid-term rehabilitation as reported by DWA (Berger et al. 2015), is conducted as follows:

- SC1: 20% increase of damage rate of private sewers;
- SC2: 20% increase of damage rate of public sewers built before the year 1980;
- SC3: 20% increase of damage rate of public sewers built after the year 1981;
- SC4: 20% increase of damage rate of all sewers.

## Results and discussion

Studies on sewer exfiltration at pipe scales indicate that the leakage process is ruled by a number of key parameters, including leakage area, depth of wastewater in the pipe, clogging layer, hydraulic permeability and soil characteristics, and hydraulic gradient from pipe surface to the groundwater (Wolf and Hötzl 2007; Karpf et al. 2009; Peche et al. 2017). These parameters describe structural and dynamic components of sewer exfiltration processes, which data demand at the catchment scale is often not available or subject to large uncertainties (Rutsch et al. 2008; Karpf and Krebs 2011; Peche 2019). The common approach for large-scale studies is so far based on mapping analysis of sewer network deteriorations (structural condition, distribution of pipes), surveying and indirectly correlating with population-based sewer discharge, or simplified assumptions of nationwide fixed rates of water loss from defective sewer pipes (e.g. Lerner et al. 1990; CIRIA 1996; Ellis and Bertrand-Krajewski 2010; Hopkins and Bain 2018; Vystavna et al. 2018). In this study, we propose an approach that combines the strength of the two abovementioned methods and utilize the advancement of knowledge from recent studies worldwide. The study aims at a generalized method which can represent the long-term mean condition of sewer exfiltration risk to the environment of large-scale urban catchments (Fig. 2). Details on results are discussed in the following sections.

### Estimation of sewer exfiltration rates

Results of calculations of sewer exfiltration are based on adjustment of parameters in Table 3. For the leakage area parameter, the default value of 0.002 m^2^ is used in all calculations following the recommendation by the study of Wolf and Hötzl (2007). The fixed water level of 0.01 and 0.02 m is selected for simplification to represent the dwf of private and public sewers for upscaled catchments (Ellis et al. 2009; Peche et al. 2017). For the thickness of colmation layer parameter, the value of 0.04 m is used for dwf and 0.01 is used for swf conditions, since at a thickness of 0.05 m the layer is considered stabilized and no exfiltration might occur (Vollertsen and Hvitved-Jacobsen 2003; Ellis et al. 2009). Meanwhile, the value of the hydraulic conductivity parameter is adjusted for validating the final exfiltration rates at single defect sites using the recommended range by Wolf and Hötzl (2007) and Karpf and Krebs (2011), due to the fact that no specific value is reported for large-scale studies for this parameter. As a result, the estimated rates of sewer leakage of public and private sewers under dwf fit well with the literature data, with the mean value of sewer leakage from private sewers being lower than from public sewers. This is justifiable considering the higher proportion of wastewater discharging into public sewers compared to such from small household sewers, which differs in the wastewater level in the pipe and the attributes of colmation layer (Ellis and Bertrand-Krajewski 2010; Wolf et al. 2012). For the swf condition, the maximum values in Wolf and Hötzl (2007) are used as the initial defaults of parameters water level in the pipe and leakage factor. These defaults resulted in the exfiltration rate of 100.22 L/day, which is rounded as 100 L/day to represent the potential mean rate of exfiltration under the rainy and storm event periods. This is 20 times higher than the estimated default rate of exfiltration under dwf (Q_ex _ pub _ dwf_ = 5 L/day), which agrees with the results from studies by Vollertsen and Hvitved-Jacobsen (2003) and Held et al. (2005), in which 20 to 56 times and 20 to 38 times higher exfiltration rates were simulated for storm events, respectively. In practice, exfiltration rates during very extreme flood event can reach of 1500 time higher values like in the case study on river Elbe, Germany, but such high rates often drop to below 1 L/day in few hours to days (e.g. Karpf and Krebs 2004; Blackwood et al. 2005). Thus, such extreme high pulses are not considered within the scope of our study, which focuses on estimating the long-term annual mean leakage rates.

At the large catchment to national scales, previous studies suggest that the exfiltration rate in Germany might be expected in the range of 0.01 to 0.2 L/s/km or even lower (Ellis et al. 2009). In the study applied to a large sewer system of 888,000 km length, Schleyer et al. (1991) estimated the mean exfiltration rate of 0.011 L/s/km. A similar rate of 0.012 L/s/km was estimated for Germany based on the study by Hoffman and Lerner (1992). The review on sewer exfiltration rates based on both direct and indirect measurement methods indicates an average exfiltration rate of less than 0.179 L/s/km in large system modelling studies (Ellis et al. 2008; Ellis et al. 2009). In this study, the rate of exfiltration of public sewers expressed as L/s/km is 0.01 and is in agreement with abovementioned publications, which are mostly conducted for public sewers in Germany. For private sewers, the amount of leakage per km sewer length is 0.002 L/s/km, which resulted in the overall sewer exfiltration for Germany of 0.004 L/s/km. Overall, the sewer exfiltration of private sewers, public sewers, and combined sewer types, are in the similar ranges of 2.0 to 2.1% when expressed as the amount of water loss from generated wastewater.

### Sources and distributions of sewer exfiltration in large urban systems

**Fig. 3:**
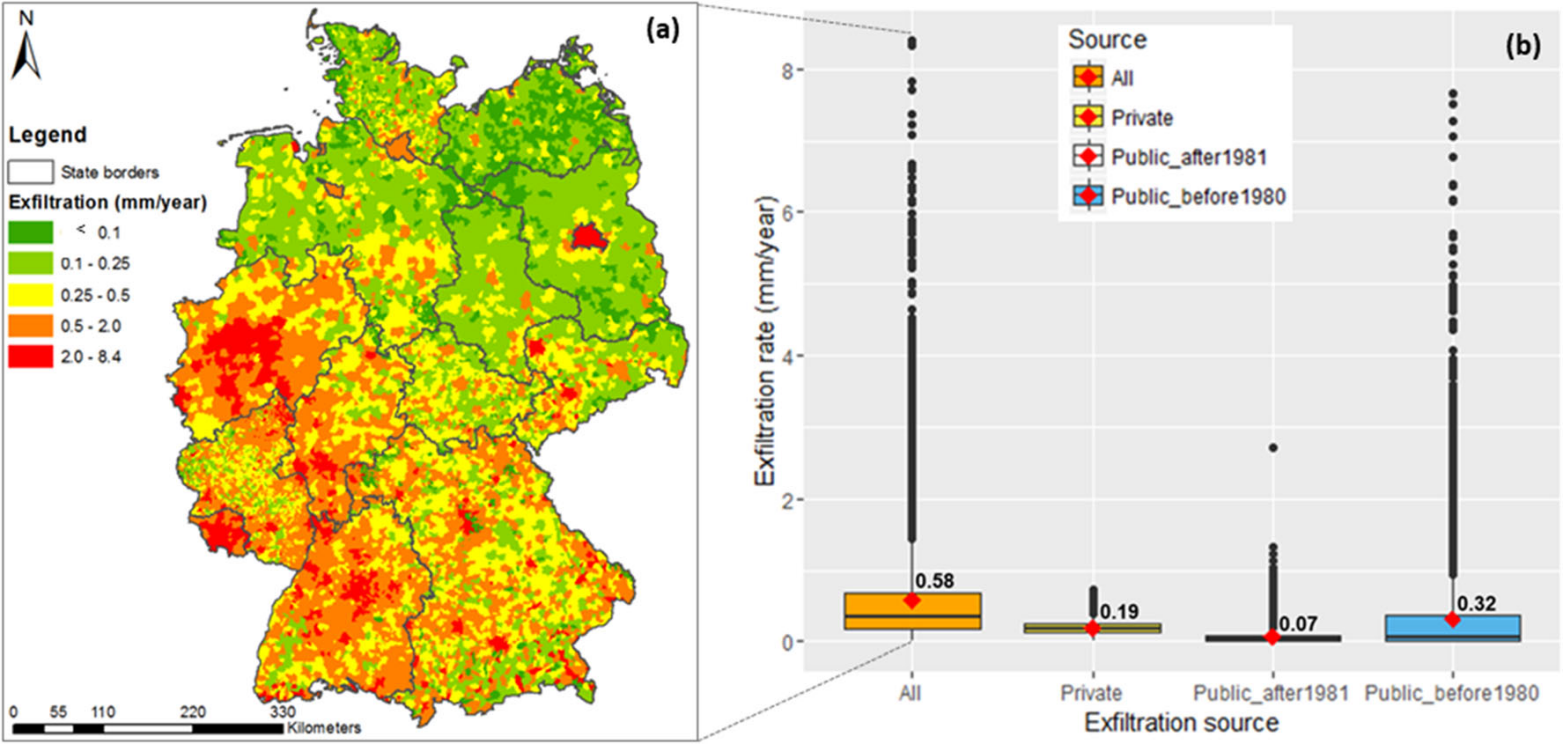
Sewer exfiltration rates in Germany: (a) spatial distribution, (b) aggregation to emission sources at municipality level (numbers on the box-plots indicate the mean exfiltration rates)

The modelled spatial distribution of sewer exfiltration from defective pipes in Germany is shown in Fig. 3(a). In general, sewer exfiltration contributes a small volume of artificial recharge to groundwater, varying from less than 0.1 to 8.4 mm/year per total municipality areas, which is equivalent to less than 1% of mean annual precipitation. Elevated exfiltration rates tend to concentrate in urbanized regions with higher shares of connected population, higher density, and often older sewer networks (Fig. 1a and Fig. 3a). Concerting the age factor of sewer systems, many studies suggest that the higher the sewer age, the greater might be the proportion of rehabilitation needs (e.g. Davies et al. 2001; Ellis and Bertrand-Krajewski 2010). Meanwhile, some studies suggest that sewers of all ages can be a potential risk to urban groundwater pollution due to diverse reasons of sewer leaks (Reynolds and Barrett 2003; Berger et al. 2015; Krönlein et al. 2015). Results of this study support both sides of arguments from literature. Dominant proportion of sewer exfiltration is caused by sewer pipes older than 40 years (Fig. 3b). In the group of younger sewers, the private pipes contribute two times higher rates of exfiltration in comparison to public sewers. A better knowledge of age distribution of private sewers might however reveal more detailed pattern of sewer leakage from this source as compared to public sewers. These outputs have an important implication for the control of defective sewers and rehabilitation plan in large urban wastewater pipe networks.

### Factors affecting the prediction of sewer exfiltration at a nationwide scale

In this study, five key model inputs have been used in sewer leakage quantification based on the available upscaled methods evolved in Germany over the recent three decades. They are readily available at municipality and aggregated levels in Germany and are defined of critical importance for the interpretation of pollutant emission estimation from sewer leakage sources. Results of the simple linear regression indicate that the wastewater amount, the number of connected inhabitants, and the number of storm events are the most important input data for modelling of sewer exfiltration (Table 4). Among these inputs, wastewater amount is the most influential data as shown by statistical results. This is in agreement with the literature which states that pipe water exfiltration tends to increase with the increases of intensity and duration of pipe flow (e.g. Schwartz 2004; Peche 2019). In contrast, changes in sewer length and sealed areas reveal a less strong impact on the variation of exfiltration rates. At municipality scale, our analyses indicate that the estimation of sewer exfiltration at large spatial scales is mainly controlled by the variation of discharges of wastewater into defective sewers, rather than by the local position of leakages in the structural network of sewer pipe length and areas of impervious surfaces. A similar finding is reported in studies by Rauch and Stegner (1994), Karpf (2012), and Peche et al. (2017) which aimed at upscaling the model approach from single-site to pipe network scales. Thus, this result is an important hint to be considered for future development of catchment to national scale urban models incorporating the emission pathway from sewer exfiltration.

**Table 4:** Analysis of important input data for modelling of sewer exfiltration at the nationwide scale

Inputs	t-Stat	*p* value	Rank
Wastewater amount	30.4	< 2e-16***	1
Inhabitant connected	− 28.7	< 2e-16***	2
Storm events	11.9	< 2e-16***	3
Sewer length	2.9	0.09***	4
Sealed area	0.1	0.29	5

*p* value—significance level (***0.001, **0.01, *0.05)

### Nutrient pollution from sewer exfiltration in large urban systems

**Fig. 4:**
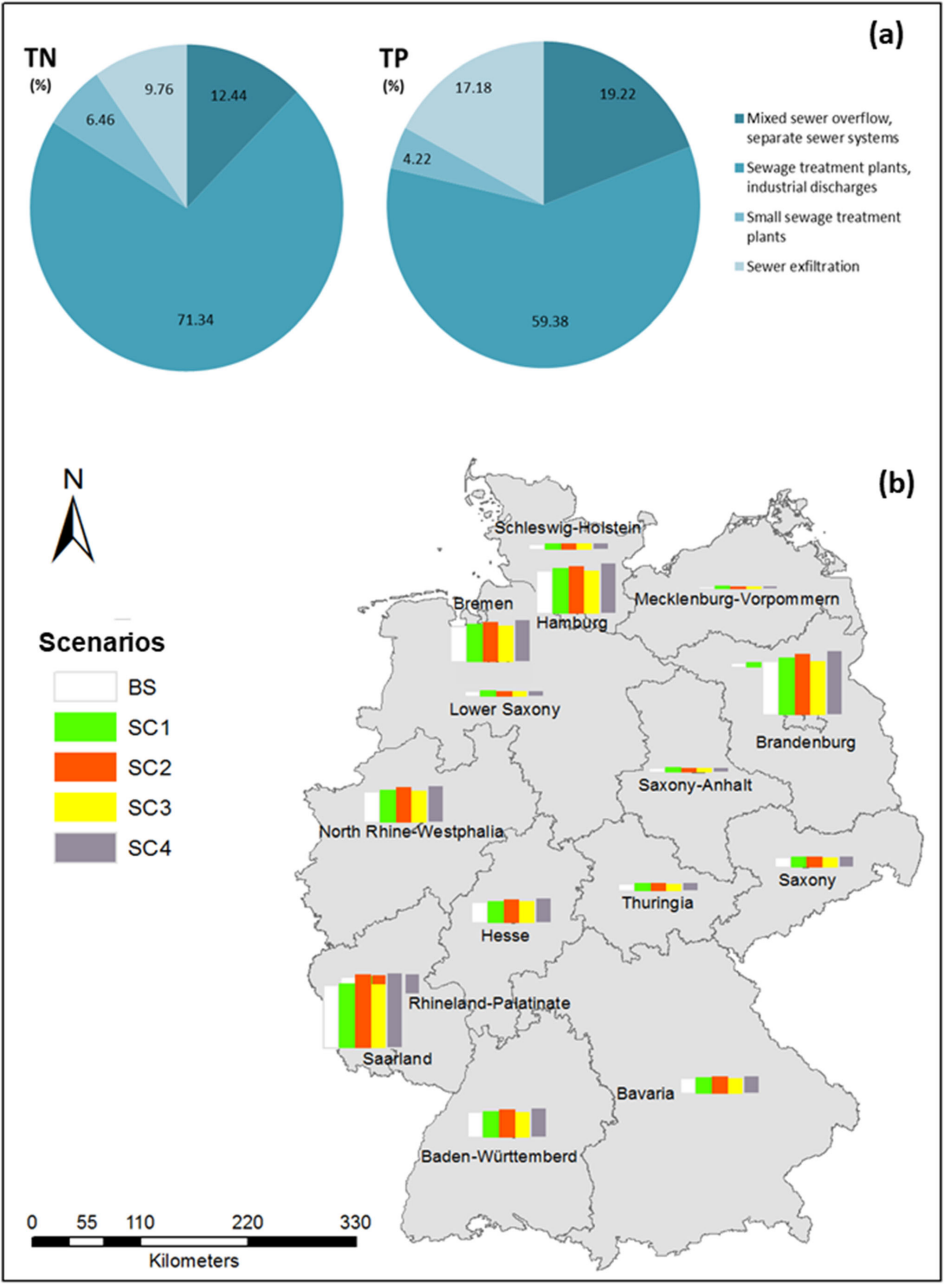
Results of scenarios on the estimated exfiltration volumes (mm/year) and loads in Germany

Adaptations and changes in national regulations which follow the on-going EU Nitrate Directive and EU Water Framework Directive require a more stringent control of nutrient pollution from diffuse emission sources of large river catchments (Scholz and Wellmer 2018; Schmidt et al. 2020). Previous studies have shown that industrial and urban wastewater sources contributed effectively to the reduction of more than 30% and 60% of phosphorus and nitrogen loads as compared to the reduction rates of only 15% and 3% to nitrogen and phosphorus respectively from agriculture in the case study of the Elbe and Rhine catchments (e.g. Behrendt et al. 2000; Sartorius et al. 2011). In another study, Behrendt et al. (2010) found out that management of diffuse sources from urban wastewater and stormwater can contribute to approximately 51% and 21.5% of the total phosphorus and total nitrogen emissions to the Elbe river catchment. These case studies reveal the importance to target primary sources from urban emission pathway in order to achieve the pollution control goal in large river systems. In this study, the preliminary results at the nationwide scale suggest that sewer exfiltration might induce around 10% and 17% of nitrate and phosphate emissions from urban systems when compared to other sources from households and wastewater treatment plants (Fig. 4). This corresponds to 12,472 tons/year and 2197 tons/year of untreated nitrate and phosphate loads being emitted to the urban groundwater from sewer exfiltration sources and stresses the importance to include sewer exfiltration as a potential significant emitter to the urban groundwater of large urban water systems.

Furthermore, results of the scenario analyses in this study indicate that areas possessing highest risk of sewer exfiltration contamination are associated with ageing deteriorated sewers. In general, increases in damage rates of sewer pipes resulted in the overall increase trend of the sewer exfiltration at the national scale for all scenarios SC1 to SC4 (Fig. 4). The highest increase of sewer exfiltration rate is caused by the increase of damage densities of public sewers older than 40 years (SC2), followed by the increase of damage densities of private sewers (SC1). Further deterioration of younger sewers constructed after the year 1981 (SC3) resulted in a minor variation of sewer exfiltration in comparison to the baseline scenario BS. Overall, the lack of remediation practice in all sewer groups might result in the overall increase of 0.11 mm/year of sewer exfiltration in the combined scenario SC4 when compared to the scenario BS, which is equivalent to the additional volume of 3750 m^3^/year of artificial recharge from sewer leakage incorporating further 2092 tons/year of nitrate and 370 tons/year of phosphate loads, respectively.

Spatially, the most impacted areas of a potentially higher sewer exfiltration risk under the scenario of no remediation actions (SC4) are located in Saarland, Berlin, Hamburg, Bremen, and North Rhine-Westphalia (Fig. 4). These are regions attributed with high proportions of very old sewer pipes nationwide; i.e. the percent of public sewers older than 40 years varies from 49 to 71% and is significantly higher than the mean value of 39% for Germany (Supplementary document S1, Fig. C). The highest exfiltration rate under SC4 scenario is modelled in Saarland, southwestern Germany, though this region has only the third highest percent of very old sewers. This can be explained by the dominant proportion of CS sewers, which have significantly higher exfiltration rates than private and BW sewers (Table 3). In particular, the percent of CSs in Saarland is above 90% when compared to the average ratio of 56% in Germany, while in the other four States this ratio varies from 26 to 63% (Table 2). Overall, this first nationwide scale overview and comparisons among different states highlight the importance to focus rehabilitation plans on very old sewers, especially on mixed sewer types which often receive high loads of wastewater from various sources.

## Conclusion

The present study represents the first German-wide estimation of wastewater losses from leaking sewers by coupling equations on sewer exfiltration quantification from local to city scales into an urban catchment model. A combination of sewer asset data and information on sewer leakage rates from literature led to the development of an algorithm to estimate the spatially differentiated sewer losses from public and private sewage systems. The results of this study indicate that the threat of sewer leakage is attributed by both private and public sewers, but more particularly from ageing pipes older than 40 years old. Effective control of the major sources of wastewater entering defective sewers, including private sewers, and a focus of rehabilitation plan on very old sewer pipes are critical points in sustainable management and pollution control of urban wastewater systems.

Future research will build on outcomes of this study by applying the proposed model framework to the scale of States and districts in order to verify the developed approach with better validation of spatially distributed parameters based on more data availability at these scales. For instance, the ‘actual’ exfiltration areas are more likely to occur in regions where the relative sewer pipe depth is above the groundwater table, as indicated by Selvakumar et al. (2004). Thus, having more complete data of both groundwater and sewer pipe depth in major catchments and cities will help to improve the precision of the spatial distribution prediction of sewer exfiltration nationwide, which are the cases of cities such as Berlin or Rastatt (e.g. Russ and Riek 2011; Wolf et al. 2012; Wunsch et al. 2018; Caradot et al. 2017). Besides, incorporating process-based approaches for important model parameters (such as wastewater level or the properties of the colmation layer) in the sewer exfiltration model to replace simplified assumptions of constant parameters, or test the time-variant leakage function instead of using the equation of a fixed sewer exfiltration rate might help to better represent the exfiltration dynamics and its spatial distribution (e.g. Peche et al. 2017; Peche 2019). This will require a more detailed identification and inventory of exfiltration susceptible areas in major cities under real sewer conditions.

## Supplementary Information

ESM 1 (DOCX 178 kb)

## Data Availability

This work is part of the interdisciplinary project ‘Agro-Environmental measures in Germany’ (AGRUM-DE). The data used in this study are available from project partners and official administrative sources within the project, and thus restrictions apply to some data sources. Data are however partly available upon reasonable request and with the permission of the project managers.

## References

[CR1] Amick RS, Burgess, EH (2000) Exfiltration in sewer systems. Report EPA/600/R-01/034, US Environmental Protection Agency, National Risk Management Research Laboratory, Ohio

[CR2] Balacco G, Iacobellis V, Portincasa F, Ragno E, Totaro V, Piccinni AF (2020). Analysis of a large maintenance journal of the sewer networks of three Apulian provinces in southern Italy. Water.

[CR3] Behrendt H, Huber P, Kornmilch M, Opitz D, Schmoll O, Scholz G, Uebe R (2000) Nutrient emissions into river basins of Germany. UBA-Texte 23-00, Federal Environmental Agency (UBA), Berlin. http://www.umweltdaten.de/publikationen/fpdf-l/1837.pdf

[CR4] Behrendt H, Venohr M, Opitz D (2010) Auswirkungen des globalen Wandels auf die Nährstoffeinträge und Frachten im Elbeeinzugsgebiet. In: Wechsung F, Hartje V, Kaden S, Behrendt H, Hansjürgens B, Gräfe P (Eds) Wirkungen des globalen Wandels auf den Wasserkreislauf im Elbegebiet - Risiken und Optionen. PIK Report, Potsdam Institute for Climate Impact Research, Potsdam

[CR5] Berger C, Falk C, Hetzel F, Pinnekamp J, Roder S, Ruppelt J (2015). Zustand der Kanalisation in Deutschland_Ergebnisse der DWA-Umfrage 2015. Korrespondenz Abwasser. Abfall.

[CR6] Bhaskar AS, Welty C, Maxwell RM, Mille AJ (2015). Untangling the effects of urban development on subsurface storage in Baltimore. Water Resour Res.

[CR7] Blackwood DJ, Ellis JB, Revitt DM, Gilmour DJ, Stainer A (2005). Factors influencing exfiltration processes in sewers. Water Sci Technol.

[CR8] Boukhemacha MA, Gogu CR, Serpescu I, Gaitanaru D, Bica I (2015). A hydrogeological conceptual approach to study urban groundwater flow in Bucharest city, Romania. Hydrogeol J.

[CR9] Burn S, Eiswirth M, Correll R, Cronin A, DeSilva D, Diaper C, Dillon P, Mohrlok U, Morris B, Rueedi J, Wolf L, Vizintin G, Vött U (2007) Urban infrastructure and its impact on groundwater contamination. In Howard, K.W.F. 2007. Urban groundwater – meeting the challenge. Selected papers from the 32nd International Geological Congress (IGC), Florence, Italy, August 2004. ISBN 0-203-94705-3

[CR10] Bütow E, Krafft H, Rüger M, Lüdecke J (2001) Gefährdungspotenzial von undichten Kanälen bei industriellen und gewerblichen Grundstücks-entwässerungsleitungen und die Ableitung von Empfehlungen zur Revitalisierung defekter Entwässerungsleitungen. Forschungsbericht,UBA-FB 000210, Umweltbundesamt

[CR11] Caradot N, Sonnenberg H, Kropp I, Ringe A, Denhez S, Hartmann A, Rouault P (2017). The relevance of sewer deterioration modelling to support asset management strategies. Urban Water J.

[CR12] Chisala BN, Lerner DN (2008). Distribution of sewer exfiltration to urban groundwater. Proc Institut Civil Eng-Water Manag.

[CR13] CIRIA (1996) Reliability of sewers in environmentally vulnerable areas. CIRIA Project Report

[CR14] Davies JP, Clarke BA, Whiter JT, Cunningham RJ (2001). Factors influencing the structural deterioration and collapse of rigid sewer pipes. Urban Water J.

[CR15] Decker J (1994). Pollution load of subsoil, groundwater and surface water by leaky sewers. Proc Hydrotop.

[CR16] Decker J, Risse A (1993) Investigations about quantitative and qualitative pollution load of subsoil, ground-water and surface water by leaky sewers. Proceedings of the 6th International Conference on Urban Storm Drainage (Marsalek J and Torno HC (eds)). Seapoint Publishing, Victoria, BC, pp1591–1596

[CR17] DeSilva D, Burn S, Tjandraatmadja G, Moglia M, Davis P, Wolf L, Held I, Vollertsen J, Williams W, Hafskjold L (2005). Sustainable management of leakage from wastewater pipelines. Water Sci Technol.

[CR18] Dohmann M, Decker J, Menzenbach B, Dohmann M (1999). Untersuchungen zur quantitativen und qualitativen Belastung von Boden, Grund- und Oberflächenwasser durch undichte Kanäle. Wassergefährdung durch undichte Kanäle: Erfassung und Bewertung.

[CR19] Ducci D (2018) An easy-to-use method for assessing nitrate contamination susceptibility in groundwater. Wiley, Hindawi. 10.1155/2018/1371825

[CR20] Dvory NZ, Kuznetsov M, Livshitz Y, Gasser G, Pankratov I, Lev O, Adar E, Yakirevich A (2018). Modeling sewage leakage and transport in carbonate aquifer using carbamazepine as an indicator. Water Res.

[CR21] Eiswirth M (2002) Bilanzierung der Stoffflüsse im urbanen Wasserkreislauf - Wege zur Nachhaltigkeit urbaner Wasserressourcen, Habilitation, University of Karlsruhe.

[CR22] Eiswirth M, Hötzl H (1997) The impact of leaking sewers on urban groundwater. In: Chilton J (Eds) Groundwater in the urban environment, ISBN-13: 978-0415434935, pp 399-404

[CR23] Eiswirth M, Held I, Wolf L, Hötzl H (2003) Assessing and improving the sustainability of urban water resources and systems – AISUWRS work package 1 Background study. Commissioned Report to the EU

[CR24] Ellis JB, Bertrand-Krajewski JL (2010). Assessing infiltration and exfiltration on the performance of urban sewer systems (APUSS).

[CR25] Ellis JB, Revitt DM, Vollertsen J, Blackwood DJ (2008) Factors influencing temporal exfiltration rates in sewer systems. In: Proceedings of the 11th International Conference on Urban Drainage (11ICUD), ISBN 978 1899796 212. Edinburgh, Scotland, UK

[CR26] Ellis J, Revitt D, Vollertsen J, Blackwood D (2009). Sewer exfiltration and the colmation layer. Water Sci Technol.

[CR27] Ennaouri I, Fuamba M (2013). New integrated condition-assessment model for combined storm-sewer systems. J Water Resour Plan Manag.

[CR28] Forschergruppe Kanalleckagen (2002) Gefährdungspotential von Abwasser aus undichten Kanälen auf Boden und Grundwasser. Arbeitsbericht der DFG-Forschergruppe an der Universität Karlsruhe. http://www.rz.uni-karlsruhe.de/~iba/kanal/zwischenbericht.pdf

[CR29] Franz T, Krebs P (2005) Statistical methods towards more efficient infiltration measurements, Proc. 10th International conference of Urban Drainage, Copenhagen, Denmark 10.2166/wst.2006.593 17120645

[CR30] Fuchs-Hanusch D, Günther M, Möderl M, Muschalla D (2015). Cause and effect oriented sewer degradation evaluation to support scheduled inspection planning. Water Sci Technol.

[CR31] Gogu CR, Boukhemacha MA, Gaitanaru D, Moraru I (2019) Interaction between city subsurface infrastructure and groundwater. In: Mannina G (eds) New trends in urban drainage modelling. Green Energy and Technology, Springer, Cham, 219–223. 10.1007/978-3-319-99867-1_36

[CR32] Hamby DM (1994). A review of techniques for parameter sensitivity analysis of environmental models. Environ Model Softw.

[CR33] Härig F, Mull R (1992). Undichte Kanalisationssysteme – die Folgen für das Grundwasser. gwf. Wasser – Abwasser.

[CR34] Harvey RR, McBean EA (2014). Predicting the structural condition of individual sanitary sewer pipes with random forests. Can J Civ Eng.

[CR35] Heinrich A (2007). Transferability of exfiltration rates from sewer systems. J Soils Sediments.

[CR36] Held I, Klinger J, Wolf L, Hötzl H, Howard K (2005). Direct measurements of exfiltration at a sewer test site under operating conditions. Matthias Eiswirth Memorial.

[CR37] Hoffman JM, Lerner DN (1992). Leak free sewers – who needs them?. Water Waste Treatment.

[CR38] Hopkins KG, Bain DJ (2018). Research note: Mapping spatial patterns in sewer age, material, and proximity to surface waterways to infer sewer leakage hotspots. Landsc Urban Plan.

[CR39] Karpf C (2012) Modellierung der Interaktion zwischen Grundwasser und Kanalisation. PhD thesis, TU Dresden

[CR40] Karpf C, Krebs P (2004) Sewers as drainage systems – quantification of groundwater infiltration. Proc 2:969–975, NOVATECH conference, Lyon, France

[CR41] Karpf C, Krebs P (2005). Application of a leakage model to assess exfiltration from sewers. Water Sci Technol.

[CR42] Karpf C, Krebs P (2011). A new sewage exfiltration model – parameters and calibration. Water Sci Technol.

[CR43] Karpf C, Traenckner J, Krebs P (2009). Hydraulic modelling of sewage exfiltration. Water Sci Technol.

[CR44] Krönlein F, Horstmeyer N, Helmreich B (2015) Zustand der öffentlichen Kanalisation in Bayern. Abschlussbericht, Bayerisches Landesamt für Umwelt

[CR45] LBM (2018) Digitales Lanbedeckungsmodell für Deutschland LBM-DE2015. Bundesamt für Kartographie und Geodäsie

[CR46] Lee DG, Roehrdanz PR, Feraud M, Ervin J, Anumol T, Jia A, Park M, Tamez C, Morelius EW, Gardea-Torresdey JL, Izbicki J, Means JC, Snyder SA, Holden PA (2015). Wastewater compounds in urban shallow groundwater wells correspond to exfiltration probabilities of nearby sewers. Water Res.

[CR47] Lerner DN, Issar AS, Simmers I (1990) Groundwater recharge - a guide to understanding and estimating natural recharge. IAH International Contributions to Hydrogeology, v8, Hannover

[CR48] Meinikmann K, Lewandowski J, Hupfer M (2015). Phosphorus in groundwater discharge a potential source for lake eutrophication. J Hydrol.

[CR49] Mohammadi MM, Najafi M, Kaushal V, Serajiantehrani R, Salehabadi N, Ashoori T (2019). Sewer pipes condition prediction models: a State-of-the-Art review. Infrastructures.

[CR50] Nakayama T, Watanabe M, Tanji K, Morioka T (2007). Effect of underground urban structures on eutrophic coastal environment. Sci Total Environ.

[CR51] Nguyen HH, Recknagel F, Meyer W, Frizenschaf J, Shrestha MK (2017). Modelling the impacts of altered management practices, land use and climate changes on the water quality of the Millbrook catchment-reservoir system in South Australia. J Environ Manag.

[CR52] Peche A (2019). Numerical modeling of pipe leakage in variably saturated soil.

[CR53] Peche A, Graf T, Fuchs L, Neuweiler I (2017). A coupled approach for the three-dimensional simulation of pipe leakage in variably saturated soil. J Hydrol.

[CR54] Peche A, Graf T, Fuchs L, Neuweiler I (2019). Physically based modeling of stormwater pipe leakage in an urban catchment. J Hydrol.

[CR55] Rauch W, Stegner T (1994). The colmation of leaks in sewer systems during dry weather flow. Water Sci Technol.

[CR56] Read GF (1996) Sewers: repair and renovation. Project Report 44, X171. Construction Industry Research and Information Association (CIRIA), London, UK

[CR57] Reynolds JH, Barrett HM (2003). A review of the effects of sewer leakage on groundwater quality. J Chart Inst Water Environ Manage.

[CR58] Rieckermann J, Borsuk ME, Sydler D, Gujer W, Reichert P (2010). Bayesian experimental design of tracer studies to monitor wastewater leakage from sewer networks. Water Resour Res.

[CR59] Roehrdanz PR, Feraud M, Lee DG, Means JC, Snyder SA, Holden PA (2017). Spatial models of sewer pipe leakage predict the occurrence of wastewater indicators in shallow urban groundwater. Environ Sci Technol.

[CR60] Rokstad MM, Ugarelli RM (2015). Evaluating the role of deterioration models for condition assessment of sewers. J Hydroinf.

[CR61] Russ A, Riek W (2011). A method to estimate groundwater depth from forest site mapping data and digital elevation models. Waldökologie, Landschaftsforschung und Naturschutz.

[CR62] Rutsch M (2006). Assessment of sewer leakage by means of exfiltration measurements and modelling tests.

[CR63] Rutsch M, Rieckermann J, Cullmann J, Ellis JB, Vollertsen J, Krebs P (2008). Towards a better understanding of sewer exfiltration. Water Res.

[CR64] Saltelli A, Ratto M, Tarantola S, Campolongo F (2006). Sensitivity analysis practices: strategies for model-based inference. Reliab Eng Syst Saf.

[CR65] Sartorius C, Hillenbrand T, Walz R (2011). Impact and cost of measures to reduce nutrient emissions from wastewater and storm water treatment in the German Elbe river basin. Reg Environ Chang.

[CR66] Schleyer R, Milde G, Milde K (1991). Development of aquifer protection policy in Germany.

[CR67] Schmidt B, Kuhn U, Trepel M, Kreins P, Zinnbauer M, Wendland F, Herrmann F, Kunkel R, Tetzlaff B, Wolters T, Venohr M, Nguyen HH (2020). Modellansatz zur Bestimmung der Nährstoffbelastung und ihrer Reduktion in allen deutschen Flussgebieten. Wasser und Abfall.

[CR68] Scholz RW, Wellmer FW (2018). Although there is no physical short-term scarcity of phosphorus, its resource efficiency should be improved. J Ind Ecol.

[CR69] Schwartz M (2004) Mikrobielle Kolmation von abwasserdurchsickerten Bodenkörpern: Nucleinsäuren zum Nachweis von Biomasse und Bioaktivität. Dissertation, University of Karlsruhe

[CR70] Selvakumar A, Field R, Burgess E, Amick R (2004). Exfiltration in sanitary sewer systems in the US. Urban Water J.

[CR71] Thomes MW, Vaezzadeh V, Zakaria MP, Bong CW (2019). Use of sterols and linear alkylbenzenes as molecular markers of sewage pollution in Southeast Asia. Environ Sci Pollut Res.

[CR72] Tscheikner-Gratl F, Caradot N, Cherqui F, Leitão GP, Ahmadi M, Langeveld JG, Gat YL, Scholten L, Roghani B, Rodríguez JP, Lepot M, Stegeman B, Heinrichsen A, Kropp I, Kerres K, Almeida MC, Bach PM, Vitry MM, Marques AS, Simões NE, Rouault P, Hernandez N, Torres A, Werey C, Rulleau B, Clemens F (2020). Sewer asset management – state of the art and research needs. Urban Water J.

[CR73] Venohr M, Nürnberg GK, Chambers PA, Guy M (2010) Predicting nutrient loads in a Canadian boreal river: applicability of a European nutrient emission model to the remote Athabasca river system. – Abstract, 14th International Conference, IWA Diffuse Pollution Specialist Group: Diffuse Pollution and Eutrophication

[CR74] Venohr M, Hirt U, Hofmann J, Opitz D, Gericke A, Wetzig A, Natho S, Neumann F, Hürdler J, Matranga M, Mahnkopf J, Gadegast M, Behrendt H (2011). Modelling of Nutrient Emissions in River Systems – MONERIS – Methods and Background. Int Rev Hydrobiol.

[CR75] Vollertsen J, Hvitved-Jacobsen T (2003). Exfiltration from gravity sewers: a pilot scale study. Water Sci Technol.

[CR76] Von Sperling DL, Behrendt H (2007) Application of the nutrient emission model MONERIS to the upper Velhas river basin, Brazil. – In: Gunkel G and Sobral M (Eds), Reservoirs and river basins management: exchange of experience from Brazil, Portugal and Germany. – Universitätsverlag, TU Berlin, pp 265–279

[CR77] Vystavna Y, Diadin D, Rossi PM, Gusyev M, Hejzlar J, Mehdlzadeh R, Huneau F (2018). Quantification of water and sewage leakages from urban infrastructure into a shallow aquifer in East Ukraine. Environ Earth Sci.

[CR78] Wolf L, Hötzl H (2007) Upscaling of laboratory results on sewer leakage and the associated uncertainty. International Contributions to Hydrogeology, Balkema, London. ISBN13 978-0-415-40745-8 (Hbk)

[CR79] Wolf L, Held I, Eiswirth M, Hötzl H (2004). Impact of leaky sewers on groundwater quality. Acta Hydrochim Hydrobiol.

[CR80] Wolf L, Eiswirth M, Hötzl H (2006). Assessing sewer–groundwater interaction at the city scale based on individual sewer defects and marker species distributions. Environ Geol.

[CR81] Wolf L, Zwiener C, Zemann M (2012). Tracking artificial sweeteners and pharmaceuticals introduced into urban groundwater by leaking sewer networks. Sci Total Environ.

[CR82] Wunsch A, Liesch T, Broda S (2018). Forecasting groundwater levels using nonlinear autoregressive networks with exogenous input (NARX). J Hydrol.

[CR83] Xu P (2004) Nutrient emissions into the Taihu Lake from the southern catchments. Report, DAAD

[CR84] Yang Y, Lerner DN, Barrett MH, Tellam JH (1999). Quantification of groundwater recharge in the city of Nottingham. Environ Geol.

[CR85] Zoppou C (2001). Review of urban storm water models. Environ Model Softw.

