# Supplementary material for: Harmonized assessment of nutrient pollution from urban systems including losses from sewer exfiltration: a case study in Germany
Source: Environ Sci Pollut Res Int. 2021 Jan 26;28(45):63878–93. doi: 10.1007/s11356-021-12440-9 (PMC8610960; doi:10.1007/s11356-021-12440-9)
Supplement: Supplementary file 1 — (DOCX 178 kb) [file 11356_2021_12440_MOESM1_ESM.docx]

**Supplementary document S1**


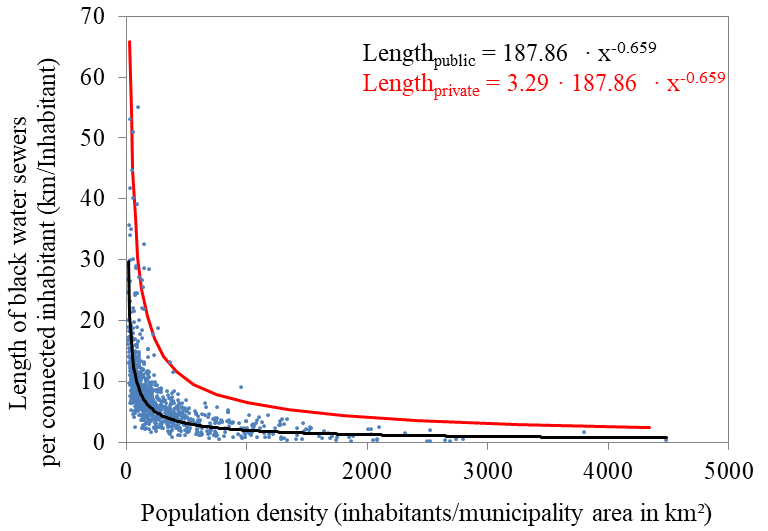


**Fig A.** Correlation between population density (x) and sewer length per connected inhabitant at municipality scale (blue dots) available for public sewers (black line) and derived for private sewers (red line) in Germany


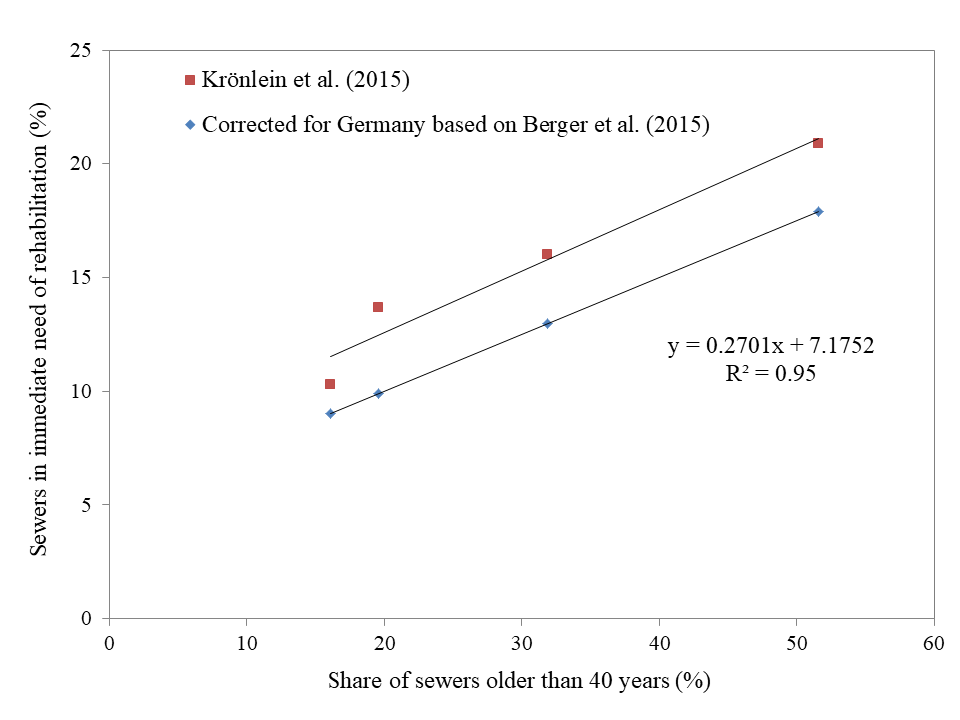


**Fig B.** Estimation of age-dependent damage rates of sewers in Germany. The equation describes the data by Krönlein et al. (2015)


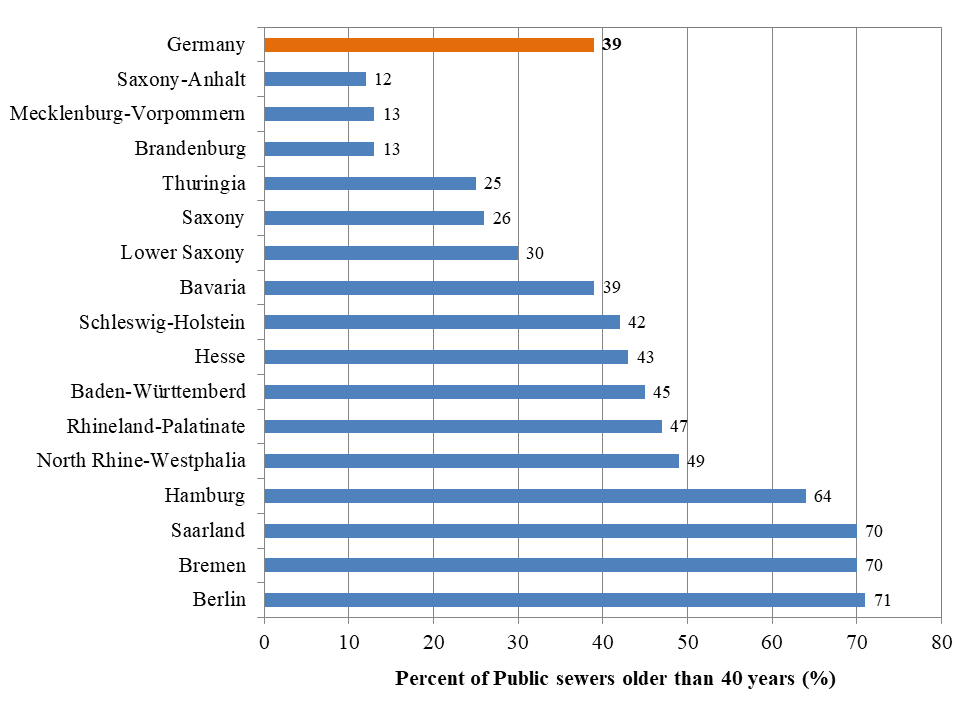


**Fig C.** Percent of sewers older than 40 years in sixteen States in Germany

**Supplementary document S2**

The following equations of the MONERIS model (Venohr et al., 2011) are used to provide some input data in this study:

$\mathrm{WW}_{\mathrm{priv}_{n}} = \mathrm{WW}_{{EW\_per\_day}_{n}}{* EW}_{\mathrm{connected}_{n}}*1000/365$ **Eq. (1)**

$\mathrm{WW}_{{pub\_CS}_{n}}=$ $\mathrm{WW}_{{pub\_CS\_bf1980}_{n}}+ \mathrm{WW}_{{pub\_CS\_af1981}_{n}}$=

$= {L\_ratio}_{{pub\_CS\_bf1980}_{n}}*\left[ \mathrm{PP}_{n}+\left( \mathrm{WW}_{\mathrm{priv}_{n}}-Q_{{ex\_priv}_{n}} \right)*{\mathrm{CS}_{\mathrm{ratio}}}_{n}/100 \right]+ {L\_ratio}_{{pub\_CS\_af1981}_{n}}*[\mathrm{PP}_{n}+\left( \mathrm{WW}_{\mathrm{priv}_{n}}-Q_{{ex\_priv}_{n}} \right)*{CS\_ratio}_{n}/100]$ **Eq. (2)**

$\mathrm{WW}_{{pub\_BW}_{n}}= \mathrm{WW}_{{pub\_BW\_bf1980}_{n}}+ \mathrm{WW}_{{pub\_BW\_af1981}_{n}}$=

$= {L\_ratio}_{{pub\_BW\_bf1980}_{n}}*\left( \mathrm{WW}_{\mathrm{priv}_{n}}-Q_{{\mathrm{ex}_{\mathrm{priv}}}_{n}} \right)*(1-{\mathrm{CS}_{\mathrm{ratio}}}_{n}/100)+ {L\_ratio}_{{pub\_BW\_af1981}_{n}}*\left( \mathrm{WW}_{\mathrm{priv}_{n}}-Q_{{ex\_priv}_{n}} \right)*(1-{\mathrm{CS}_{\mathrm{ratio}}}_{n}/100)$ **Eq. (3)**

$\mathrm{WWP}_{\mathrm{priv}_{n}}=1.95{* EW}_{\mathrm{connected}_{n}}*365/1000$ **Eq. (4)**

$\mathrm{WWP}_{{pub\_CS}_{n}}=\left[ \left( \mathrm{WWP}_{\mathrm{priv}_{n}}-P_{{loss\_priv}_{n}} \right)*{\mathrm{CS}_{\mathrm{ratio}}}_{n}/100 \right]+[\frac{(37+2.5*100)}{1000}*\mathrm{Sealed}_{n}*{\mathrm{CS}_{\mathrm{ratio}}}_{n}]$ **Eq. (5)**

$\mathrm{WWP}_{{pub\_BW}_{n}}=\left( \mathrm{WWP}_{\mathrm{priv}_{n}}-P_{{loss\_priv}_{n}} \right)*(1-{\mathrm{CS}_{\mathrm{ratio}}}_{n}/100$ **) Eq. (6)**

where:

$\mathrm{WW}_{\mathrm{priv}_{n}}$ = amount of wastewater generated from inhabitants (m³/year),

$\mathrm{WW}_{{pub\_CS}_{n}}$ = amount of wastewater generated from combined sewers (m³/year),

$\mathrm{WW}_{{pub\_BW}_{n}}$ = amount of wastewater generated from separate sewers (m³/year),

$\mathrm{WW}_{{pub\_CS\_bf1980}_{n}}$ = amount of wastewater generated from combined sewers built before 1980 (m³/year),

$\mathrm{WW}_{{pub\_CS\_af1981}_{n}}$ = amount of wastewater generated from combined sewers built after 1981 (m³/year),

$\mathrm{WW}_{{pub\_BW\_bf1980}_{n}}$ = amount of wastewater generated from separate sewers built before 1980 (m³/year),

$\mathrm{WW}_{{pub\_BW\_af1981}_{n}}$ = amount of wastewater generated from separate sewers built after 1981 (m³/year),

${L\_ratio}_{{pub\_CS\_bf1980}_{n}}$ = ratio of pipe length of combined sewers built before 1980 (km/km),

${L\_ratio}_{{pub\_CS\_af1981}_{n}}$ = ratio of pipe length of combined sewers built after 1981 (km/km),

${L\_ratio}_{{pub\_BW\_bf1980}_{n}}$ = ratio of pipe length of separate sewers built before 1980 (km/km),

${L\_ratio}_{{pub\_BW\_af1981}_{n}}$ = ratio of pipe length of separate sewers built after 1981 (km/km),

$\mathrm{WW}_{{EW\_per\_day}_{n}}$ = amount of wastewater generated per inhabitant per day (L/Inhabitant/day),

$\mathrm{WWP}_{\mathrm{priv}_{n}}$ = P load exfiltrated from private sewers (tons/year),

$\mathrm{WWP}_{{pub\_CS}_{n}}$ = P load exfiltrated from combined sewers (tons/year),

$\mathrm{WWP}_{{pub\_BW}_{n}}$ = P load exfiltrated from separate sewers (tons/year),

$\mathrm{EW}_{\mathrm{connected}_{n}}$ = connected inhabitants per municipality (Inhabitant),

$\mathrm{PP}_{n}$ = amount of precipitation (m³/year),

${CS\_ratio}_{n}$ = percent of combined sewer in the total public sewer length (%), and

$\mathrm{Sealed}_{n}$ = total sealed areas per municipality (km²).

Similar functions for nitrate as for phosphate load calculations (Eq. 4, 5, 6) were applied for public CSs and BWs, and private sewers, with a distinction on the inhabitant-specific coefficients. In particular, inhabitant-specific emissions of 1.95 g/inhabitant/day and 11 g/inhabitant/day were used for phosphate and nitrate load estimations in MONERIS, respectively.
